# The rollout of paediatric dolutegravir and virological outcomes among children living with HIV in Mozambique

**DOI:** 10.4102/sajhivmed.v25i1.1578

**Published:** 2024-07-31

**Authors:** Ivete Meque, Nicole Herrera, Amâncio Nhangave, Dórcia Mandlate, Rui Guilaze, Ana Tambo, Abdul Mussa, Nilesh Bhatt, Michelle M. Gill

**Affiliations:** 1 Elizabeth Glaser Pediatric AIDS Foundation, Maputo, Mozambique; 2 Elizabeth Glaser Pediatric AIDS Foundation, Washington DC, United States of America; 3 Núcleo Provincial de Pesquisa de Gaza, Direcção Provincial de Saúde de Gaza, Xai-Xai, Mozambique; 4 Núcleo Provincial de Pesquisa de Inhambane, Direcção Provincial de Saúde de Inhambane, Inhambane, Mozambique

**Keywords:** antiretroviral treatment, children, paediatric dolutegravir, viral suppression, Mozambique

## Abstract

**Background:**

In 2022, Mozambique introduced Dolutegravir 10mg (pDTG), as part of paediatric antiretroviral therapy for children weighing < 20 kg. Understanding real-world challenges during national rollout can strengthen health systems in resource-limited settings.

**Objectives:**

We described the transition rate to, and new initiation of, pDTG, viral load suppression (VLS) post-pDTG, and factors associated with VLS among children living with HIV.

**Method:**

We conducted a retrospective cohort study involving children aged < 9 years and abstracted data from clinical sources. We used logistic regression to assess VLS and pDTG initiation predictors.

**Results:**

Of 1353 children, 1146 initiated pDTG; 196 (14.5%) had no recorded weight. Post-pDTG switch, 98.9% (950/961) of children maintained the same nucleoside reverse transcriptase inhibitor backbone. After initiating Abacavir/Lamivudine+pDTG, 834 (72.8%) children remained on the regimen, 156 (13.6%) switched off (majority to Dolutegravir 50mg), 22 (1.9%) had ≥ 2 anchor drug switches; 134 (11.7%) had no documented follow-up regimen. Factors associated with pDTG initiation or switch were younger age (adjusted odds ratio [AOR] = 0.71 [0.63–0.80]) and a recorded weight (AOR = 55.58 [33.88–91.18]). VLS among the 294 children with a viral load (VL) test after ≥ 5 months post-pDTG was 75.5% (*n* = 222/294). Pre-pDTG VLS rate among treatment-experienced children was 56.5% (*n* = 130/230). Factors associated with VLS were older age (AOR = 1.18 [1.03–1.34]) and previous VLS (AOR = 2.27 [1.27–4.06]).

**Conclusion:**

Most eligible children initiated pDTG per guidelines, improving post-pDTG VLS. Challenges included unexplained switches off pDTG after initiation, low VL coverage and inadequate documentation in clinic records.

**What this study adds:** This study adds to limited real-world evidence on the challenges to rollout of paediatric formulations that can inform opportunities to strengthen HIV services for children living with HIV in resource-limited settings.

## Introduction

Children aged 0 to 14 years account for approximately 5% of all people living with HIV worldwide, with the majority (88%) residing in sub-Saharan Africa.^1^ Despite progress in access to effective antiretroviral therapy (ART) across the globe, children living with HIV (CLHIV) continue to have marked disparities in access to life-saving and optimal paediatric ART and in reaching viral load suppression (VLS). In 2021, of the estimated 1.68 million CLHIV aged 0 to 14 years, only 52% received ART^1^ compared to 76% of adults living with HIV, both below the 95% global target of treatment coverage.^2^ The VLS rate among CLHIV is also suboptimal. A meta-analysis of studies among CLHIV from low- and middle-income countries found a VLS rate within 12 months of ART initiation of only 72.7%.^3^

With an estimated HIV prevalence in 2021 of 12.4% among people aged 15 years to 49 years,^4^ Mozambique is currently one of the top 10 countries with the highest HIV prevalence worldwide.^5^ CLHIV constitute 6% of all people living with HIV in the country. In 2022, the Mozambique Ministry of Health (MoH) reported that only 73% of CLHIV received ART, of which 71% were virally suppressed, far below the 95% global target.^6^

Dolutegravir (DTG) is an integrase strand transfer inhibitor recommended as first-line ART for children and adults in combination with a backbone of two nucleoside reverse transcriptase inhibitors (NRTI).^7^ DTG-based ART is associated with better virologic outcomes than other antiretroviral drugs.^8,9^ As demonstrated by clinical trials in adults and children, DTG-based regimens have faster VLS than protease inhibitors (PI) and non-nucleoside reverse transcriptase inhibitors (NNRTI).^10,11,12,13^

Following guidelines from 2018 on the use of DTG as first- and second-line treatment for CLHIV weighing ≥ 20 kg, in 2019, the World Health Organization (WHO) updated these guidelines^7^ recommending the use of paediatric DTG (pDTG) 10 mg dispersible tablets for younger children weighing < 20 kg. After revising its national guidelines, Mozambique introduced pDTG in June 2021, with its subsequent rollout in late February 2022.^14^

According to national guidelines, all Mozambican children initiating ART and switching to new antiretroviral (ARV) regimens, including pDTG-based regimens, should undertake the first VL monitoring 6 months after ART initiation and at every 12 months thereafter if the VL result is < 1000 copies/mL. Children with VL ≥ 1000 copies/mL, should repeat VL 3 months after three enhanced counselling sessions.^15^ Prior national guidelines dated from 15 October 2020 recommended that children switch to or initiate ART with Abacavir (ABC) or Zidovudine (AZT)/Lamivudine (3TC)+Lopinavir/Ritonavir (LPV/r) regimen. After pDTG national rollout in February 2022, all children from 4 weeks of age and weighing < 20 kg (3 kg – 19.9 kg) regardless their ART regimen or VL result, were recommended to switch to or initiate ART with ABC/3TC+pDTG and then switch to ABC/3TC+ DTG 50 mg when they reached 20 kg.^16^

There is limited real-world evidence of pDTG national rollout targeting CLHIV in resource-limited settings. This study aims to describe programme rollout, including the transition rate to and new initiation of pDTG, VLS rates post-pDTG and factors associated with VLS among Mozambican CLHIV.

## Research methods and design

### Study design, setting and population

This is a retrospective observational cohort study using routinely collected data, conducted in 16 health facilities (10 in Gaza and 6 in Inhambane provinces) supported by Elizabeth Glaser Pediatric Foundation (EGPAF) under the Unitaid-funded Optimal project in Mozambique. Sites in rural and urban areas were purposively selected based on ≥ 60% viral load (VL) coverage among children.

Study participants were children < 9 years of age as of 31 March 2022, weighing < 20 kg or with an unknown weight, or weighing ≥ 20 kg but receiving pDTG, and newly or currently enrolled in HIV care and treatment services at a study site. We chose a 9-year age threshold because based on the Mozambique reference guide on growth tables for boys and girls from 0 to 18 years of age, both boys and girls as old as 8 years could be < 20 kg.^17,18^ WHO standards define 19 kg as the minimum weight for children 8 years – 9 years.^19^

### Data collection

We reviewed records of all children < 9 years of age as of 31 March 2022 receiving HIV services at study sites to determine eligibility for pDTG and enrolment into the study. Children < 9 years were excluded if they weighed ≥ 20 kg and never started or switched to pDTG. We collected data between October 2022 and November 2022 for a study period from March 2022 to September 2022. Data were extracted from the patient-level electronic medical database (OpenMRS) to a study-specific database in ODK-X. To the extent possible, we triangulated these data and addressed missing information in OpenMRS by using other electronic sources, such as pharmacy and laboratory records, and paper-based medical files. We captured demographic and relevant clinic information and follow-up outcomes for children weighing < 20 kg and who did not initiate pDTG during the study period, as they were available.

### Data analysis

The primary outcomes were a switch to pDTG (a switch to pDTG with previous exposure to any non-DTG-based regimen or DTG 50 mg) or a new initiation on pDTG without previous ART exposure. We define VLS on pDTG as HIV viral load < 1000 copies/mL ≥ 5 months after pDTG initiation or switch. Five months was used as the cut-off to maximise the number of children contributing to the VL outcome while reflecting a clinically significant duration. Clients were considered active in care if there was not another follow-up outcome documented: treatment suspension, loss to follow-up, transfer out, or death.

In all analyses, we included covariates such as demographic characteristics (sex and age at HIV diagnosis, age at ART and age at pDTG initiation), and clinical covariates that included ART initiation regimen, weight and time on ART at pDTG initiation, previous ART experience and date of pDTG initiation.

We analysed data using SAS (version 9.4) with a statistical significance set at *P* < 0.05. We calculated numbers and percentages for categorical variables and presented median and interquartile ranges (IQRs) for continuous variables. We use a chi-square test for categorical variables and a Wilcoxon-Mann-Whitney test for continuous variables for statistical comparisons. We used odds ratios (OR) and 95% confidence intervals (CI) for both unadjusted and adjusted logistic regression models to assess predictors of pDTG switch or post-pDTG VLS.

### Ethical considerations

This study was approved with a waiver of the requirement for individual consent, as only routinely collected programme data were used, by the National Bioethics Committee for Health of Mozambique on 24 December 2019 (IRB no. 00002657; reference no. 656/CNBS/19) and Advarra in the United States on 26 February 2020 (IRB no. 00000971; Protocol Number Pro00042176).

## Results

### Baseline characteristics

**FIGURE 1:**
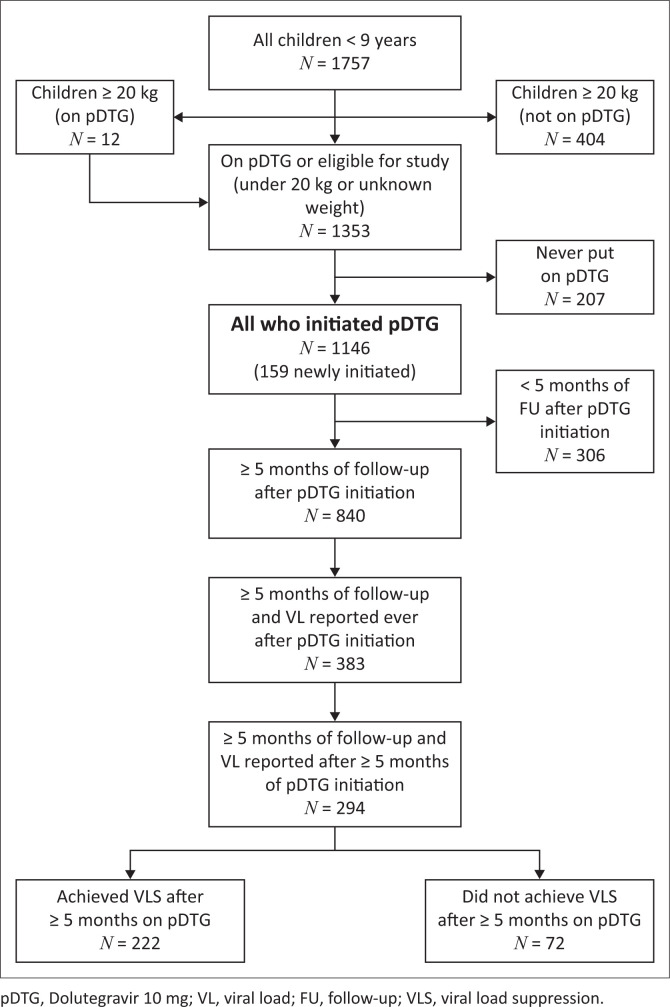
Flowchart of study screening, enrolment and follow-up.

Among 1353 children eligible for pDTG initiation, 84.7% transitioned to or initiated ABC/3TC+pDTG, leading to a final study cohort of 1146 children (Figure 1). Many children started ABC/3TC+pDTG in the first month (March 2022) after the formulation was made available at site level. A total of 207 children under 20 kg or with an unknown weight never initiated ABC/3TC+pDTG. The final cohort also includes 12 children who weighed ≥ 20 kg and started ABC/3TC+pDTG. Of the 1146 children starting ABC/3TC+pDTG, 987 (86%) were ART experienced. Most children in the cohort (*n* = 840, 73%) had ≥ 5 months of follow-up after ABC/3TC+pDTG initiation.

The median age of those ever on pDTG during the study was significantly younger, 4.1 years (interquartile range [IQR]: 2.4–5.7), compared to those who were not, 7.2 years (IQR: 5.9–8.2) (*P* < 0.0001); 53.0% of all children were female (Table 1a and Table 1b). Overall, 98.9% of treatment-experienced children (*n* = 950/961) maintained the same backbone (ABC/3TC) prior to switch and 11 (1.1%) had a backbone switch when switched to the pDTG regimen. Of those, eight had a switch from AZT+3TC to ABC+3TC, two had a switch from AZT+3TC+ABC to ABC+3TC, and one had a switch from TDF+3TC to ABC+3TC. Among all children starting ABC/3TC+pDTG, those newly initiating were significantly younger compared to children switching to ABC/3TC+pDTG: median age 1.7 years (IQR: 0.8–4.3) vs 4.3 years (IQR: 2.8–5.8), *P* < 0.0001 (data not shown). Most children who switched to ABC/3TC+pDTG transitioned from LPV/r-based (50.2%) or NVP-based (31.3%) ART regimens. Those not initiating ABC/3TC+pDTG were more likely to have been on ART for ≥ 2 years (79.5% vs. 54.3%, *P* < 0.0001). Among those not initiating ABC/3TC+pDTG, more than half (59.2%) of children had initiated ART with an NVP-based regimen; 31.3% had initiated a LPV/r-based regimen. Of those not started ABC/3TC+pDTG, almost a quarter (*n* = 48, 23.2%) weighed < 20 kg and nearly three-quarters did not have weight data (*n* = 159, 76.8%). At the end of study follow-up, most children were still in care; children who initiated ABC/3TC+pDTG were more likely to be in care than those who did not (94.1% vs. 85.5%, *P* < 0.0001). Of those eligible children who did not initiate ABC/3TC+pDTG (*n* = 207), most were receiving a regimen with DTG 50 mg (*n* = 128, 62.8%); of these, 124 children had an unknown weight and a median age of 7.8 years (IQR: 6.6–8.3).

**TABLE 1a:** Baseline characteristics after switch to ABC/3TC+pDTG initiation or by March 2022.

Variable	Not initiated ABC/3TC+pDTG (*n* = 207)		Initiated ABC/3TC+pDTG (*n* = 1146)		Total (*N* = 1353)		*P*
	Median	IQR	Median	IQR	Median	IQR	
Age at pDTG initiation OR on 31 March 2022	7.2	5.9–8.2	4.1	2.4–5.7	4.6	2.7–6.3	< 0.0001*

ABC, Abacavir; IQR, interquartile range; pDTG, Dolutegravir 10 mg; 3TC, Lamivudine.

* , values significant at *P* < 0.05.

**TABLE 1b:** Baseline characteristics after switch to ABC/3TC+pDTG initiation or by March 2022.

Variable	Not initiated ABC/3TC+pDTG (*n* = 207)		Initiated ABC/3TC+pDTG (*n* = 1146)		Total (*N* = 1353)		*P*
	*n*	%	*n*	%	*n*	%	
**Sex**							0.2400
Male	105	50.7	531	46.3	636	47.0	-
Female	102	49.3	615	53.7	717	53.0	-
**Time on ART at pDTG initiation OR time on ART by 31 March 2022**							< 0.0001*
< 6 months	15	7.3	255	22.4	270	20.1	-
6 months to < 1 year	11	5.4	74	6.5	85	6.3	-
1 to < 2 years	16	7.8	191	16.8	207	15.4	-
≥ 2 years	163	79.5	619	54.3	782	58.2	-
*Missing ART initiation date*	*2*	-	*7*	-	*9*	-	-
**ART anchor drug at ART initiation**							< 0.0001*
EFV	5	2.5	18	1.6	23	1.7	-
NVP	119	59.2	358	31.3	477	35.4	-
ATV	1	0.5	10	0.9	11	0.8	-
DRV	0	0.0	1	0.1	1	0.1	-
LPV/r	63	31.3	575	50.2	638	47.4	-
DTG 50 mg	13	6.5	0	0.0	13	1.0	-
pDTG (newly initiated on treatment)	-	-	159	13.9	159	11.8	-
3 NRTI (AZT+3TC+ABC)	0	0.0	3	0.3	3	0.2	-
DRV/r + RAL	0	0.0	1	0.1	1	0.1	-
It could not be determined	0	0.0	20	1.8	20	1.5	-
*Missing*	*6*	-	*1*	-	*7*	-	-
**ART anchor drug immediately prior to pDTG transition or current regimen on 31 March 2022**							< 0.0001*
None (newly initiated on pDTG)	0	0.0	159	14.0	159	11.8	-
EFV	0	0.0	6	0.5	6	0.5	-
NVP	0	0.0	7	0.6	7	0.5	-
ATV	0	0.0	1	0.1	1	0.1	-
LPV/r	37	37.2	917	80.4	993	73.9	-
DTG 50 mg	62	62.8	28	2.5	156	11.6	-
3 NRTI (AZT+3TC+ABC)	0	0.0	2	0.2	2	0.2	-
Could not be determined	0	0.0	20	1.8	20	1.5	-
*Missing*	*3*	-	*6*	-	*9*	-	-
**pDTG initiation date**							
January to 31 March 2022	-	-	628	54.8	628	54.8	-
On or after 01 April 2022	-	-	518	45.2	518	45.2	-
**Weight at pDTG initiation or most recent by 31 March 2022**							< 0.0001*
< 20 kg	48	23.2	1097	95.7	1145	84.6	-
≥ 20 kg	-	-	12	1.1	12	0.9	-
Unknown weight	159	76.8	37	3.2	196	14.5	-
**Outcome by 30 September 2022**							< 0.0001*
Discontinuation	2	1.0	1	0.1	3	0.2	-
Lost to follow-up	2	1.0	1	0.1	3	0.2	-
Transferred out	21	10.1	57	5.0	78	5.8	-
Death	5	2.4	9	0.8	14	1.0	-
Active in care	177	85.5	1078	94.1	1255	92.8	-

ART, antiretroviral therapy; NRTI, nucleoside reverse transcriptase inhibitors; EFV, Efavirenz; NVP, Nevirapine; ATV, Atazanavir; DRV, Darunavir; DRV/r, Darunavir/Ritonavir; LPV/r, Lopinavir/Ritonavir; DTG, Dolutegravir; pDTG, Dolutegravir 10 mg; RAL, Raltegravir; AZT, Zidovudine; 3TC, Lamivudine; ABC, Abacavir.

* , values significant at *P* < 0.05.

### ARV switching

Of the 1146 on ABC/3TC+pDTG, 134 (11.7%) had no follow-up regimen documented (Table 2). Of these children, 53 (39.6%) were not initiated until August or September 2022 (near the end of data collection). This group also accounts for 54.4% (*n* = 33/59) of ART discontinuation, loss to follow-up, transfers, and 55.6% (*n* = 5/9) of the deaths. A total of 834 (72.8%) children had no change in regimen after starting ABC/3TC+pDTG; all had ≥ 1 visit within the study follow-up period after they were dispensed this regimen (Table 2). After initiating ABC/3TC+pDTG, 178 children switched off this regimen during the follow-up period, the majority of whom had ≥ 1 changes and switched to DTG 50 mg (*n* = 159, 89.3%) and were ≥ 5 years old (86.2%). Of the 159 children who switched to DTG 50 mg, 13 were < 20 kg or had an unknown weight; five of these children switched back to ABC/3TC+pDTG by the study’s end. Twenty-two children had ≥ 2 switches; 17 transitioned from pDTG to another regimen and back to ABC/3TC+pDTG, while one switched from pDTG to LPV/r and ended on DTG 50 mg. Three children had three regimen switches, alternating between ABC/3TC+pDTG and DTG 50 mg with DTG 50 mg as their final regimen. One child with four switches started and ended on ABC/3TC+pDTG.

**TABLE 2:** Antiretroviral therapy switches and substitutions by age band after pDTG initiation.

Variable	Children < 5 years (*n* = 721)		Children ≥ 5 to < 10 years (*n* = 425)		Total (*N* = 1146)	
	*n*	%	*n*	%	*n*	%
**A. Overall number ART changes**						
No follow-up regimen documented	102	14.1	32	7.5	134	11.7
No change	585	81.1	249	58.6	834	72.8
1 change (all from pDTG)	25	3.5	131	30.8	156	13.6
2 changes	8	1.1	10	2.4	18	1.6
3 changes	-	-	3	0.7	3	0.3
4 changes	1	0.1	-	-	1	0.1
**B. Children with one change from pDTG**	25	-	131	-	156	-
To DTG 50 mg	17	68.0	128	97.7	145	92.9
To another (unknown) regimen	8	32.0	3	2.3	11	7.1

ART, antiretroviral therapy; pDTG, Dolutegravir 10 mg; DTG, Dolutegravir.

Of the 159 newly initiated children, 62 (39.0%) had no data after pDTG initiation, 82 (51.6%) remained on pDTG and 15 (9.4%) had ≥ 1 switch. Of those without any follow-up visit information, 37 (59.7%) were initiated in August 2022 or September 2022 (near the end of the study period).

Table 3 presents predictors associated with switching to pDTG among ART-experienced children only. The only factors independently associated with pDTG switch were younger age and having a weight recorded in clinic records, which informs the determination of formulation eligibility.

**TABLE 3:** Model of pDTG switch using logistic regression.

Variable	Unadjusted OR (95% CI)	Adjusted OR (95% CI)	*P*
**Age (continuous)**	0.52 (0.47–0.58)	0.71 (0.63–0.80)	< 0.0001*
**Sex**			
Male	Ref	Ref	-
Female	1.22 (0.90–1.65)	1.40 (0.87–2.23)	0.16
**Time on ART at pDTG switch or by 31 March 2022**			
< 6 months	Ref	-	-
6 months – < 1 year	1.03 (0.45–2.37)	-	-
1 year – < 2 years	1.83 (0.87–3.85)	-	-
≥2 years	0.58 (0.33–1.03)	-	-
**Weight recorded**			
No	Ref	Ref	-
Yes	92.94 (58.07–148.75)	55.58 (33.88–91.18)	< 0.0001*

pDTG, Dolutegravir 10 mg; OR, odds ratio; ART, antiretroviral therapy; CI, confidence interval; Ref, reference.

* , values significant at *P* < 0.05.

### Virological outcomes

Table 4a and Table 4b present VLS data by children switching to pDTG from another regimen and those newly initiating pDTG. Among those who transitioned to pDTG, VL coverage was 45.9%. Treatment-experienced children were more likely to have VL monitoring performed after the switch than those newly initiated on pDTG after initiation (47.9% vs. 18.6%, *P* < 0.0001), but treatment-experienced children were also more likely to have received the drug for ≥ 5 months (79.6% vs. 37.1%, *P* < 0.0001). In total, 26.0% of children on pDTG for ≥ 5 months had a VL result and, thus, contributed data to the VLS outcome. Overall median months on pDTG at the time of VL collection was 6.0 months (5.9 months – 6.1 months). The VLS rate was higher for treatment-naïve children, but the numbers were small (*n* = 9), and the difference between the two treatment groups was not statistically different (*P* = 0.34). A total of 294 children (of whom 285 were treatment-experienced) received VL testing after ≥ 5 months on pDTG and the VLS rate was only calculated among these children; 222 children were virally suppressed with a VLS rate of 75.5% (*n* = 222/294). Among treatment-experienced children with a pDTG VL result ≥ 5 months and an available VL prior to switch to pDTG, their pre-pDTG VLS rate was 56.5% (*n* = 130/230).

**TABLE 4a:** Viral load results after ≥ 5 months on pDTG, by treatment experience.

Variable	Treatment-experienced on pDTG		Newly initiated on pDTG		Overall		*P*
	Median	IQR	Median	IQR	Median	IQR	
Months on pDTG at VL	6.0	5.9–6.1	6.0	5.9–6.1	6.0	5.9–6.1	0.59

pDTG, Dolutegravir 10 mg; VL, viral load; IQR, interquartile range.

* , values significant at *P* < 0.05.

**TABLE 4b:** Viral load results after ≥ 5 months on pDTG, by treatment experience.

Variable	Treatment-experienced on pDTG		Newly initiated on pDTG		Overall		*P*
	*n*	%	*n*	%	*N*	%	
Children on pDTG	987	86.1	159	13.9	1146	-	-
Children on pDTG ≥ 5 months	776	79.6	59	37.1	835	73.9	< 0.0001*
Children on pDTG with VL	372	47.9	11	18.6	383	45.9	< 0.0001*
Children on pDTG with VL ≥ 5 months after initiation or switch	285	76.6	9	81.8	294	76.8	0.69
**pDTG VL result**	-	-			-	-	0.34
Suppressed	214	75.1	8	88.9	222	75.5	-
Unsuppressed	71	24.9	1	11.1	72	24.5	-
**Pre-pDTG VL result**	-	-	-	-	-	-	-
Suppressed	130	56.5	-	-	130	56.5	-
Unsuppressed	100	43.5	-	-	100	100.0	-

pDTG, Dolutegravir 10 mg; VL, viral load.

* , values significant at *P* < 0.05.

Table 5 presents predictors associated with VLS among children on pDTG. The only factors independently associated with VLS were older age and having a previously suppressed VL. Switching from another ARV regimen was not associated with VLS, but newly initiated children were likely to be younger and would not have had a pre-pDTG VL collected.

**TABLE 5:** Model of viral load suppression after ≥ 5 months on pDTG using logistic regression.

Variable	Unadjusted OR (95% CI)	Adjusted OR (95% CI)	*P*
**Age (continuous)**	1.19 (1.05–1.35)	1.18 (1.03–1.34)	0.02*
**Sex**			
Male	Ref	-	-
Female	1.30 (0.79–2.14)	-	-
**Previous ART**			
Yes: treatment-experienced	Ref	-	-
No: newly initiated	2.78 (0.34–22.54)	-	-
**Pre-pDTG VLS**			
Unsuppressed	Ref	Ref	-
Suppressed	2.38 (1.34–4.25)	2.27 (1.27–4.06)	0.01*
No pre-pDTG VL	1.43 (0.75–2.73)	1.64 (0.85–3.19)	0.14

pDTG, Dolutegravir 10 mg; OR, odds ratio; ART, antiretroviral therapy; VLS, viral load suppression; CI, confidence interval; VL, viral load; Ref, reference.

* , values significant at *P* < 0.05.

## Discussion

This is one of the few studies providing real-world evidence of pDTG national rollout, including post-transition virological outcomes in a sample of Mozambican children younger than 9 years. Most eligible children transitioned or initiated pDTG per national guidelines, and after ≥ 5 months post transition, slightly over three-quarters of children were virally suppressed. However, our study also identified challenges during the national rollout of this new ARV formulation. These included switches off pDTG after starting, VL monitoring gaps pre and post transition to pDTG, and incomplete documentation of ARV regimens and weight to determine drug eligibility. We also observed that some potentially eligible children did not switch to pDTG. A greater understanding of these challenges presents opportunities for strengthening health systems, primarily those in sub-Saharan Africa.

We found that most children initiated pDTG immediately after the formulation was made available at site level in March 2022, which aligns with the release of national pDTG guidelines. As expected, most eligible children who switched to pDTG were ART experienced. This is consistent with results from the rollout of the DTG 50 mg in the same 16 study sites, finding that about 96% of children who transitioned to DTG 50 mg were ART-experienced.^20^ In our study, children of younger ages were more likely to initiate ART with pDTG than older children.

Overall, 15% of potentially eligible children < 9 years (*n* = 207) did not switch to pDTG after the national pDTG rollout. Of this group, 124 (59.9%) children had an unknown weight, but were all on DTG 50 mg and were more likely to be older, with a median age of 7.8 years (IQR: 6.6–8.3). If we assume this group was ≥ 20 kg based on their older age, and thus not eligible for pDTG and on the appropriate DTG formulation, we can estimate the rate of pDTG transition among potentially eligible children is between 85% and 93%. However, we also found that almost a quarter (*n* = 48) of children weighing < 20 kg did not transition to pDTG. Concerns about pDTG supply and an overstock of LPV/r may have also delayed transition among some ART-experienced children until late June 2022 – August 2022 and is likely to explain these findings. Also, we cannot ignore issues of the stock management system at sub-national levels that may potentially result in stockouts of ARVs, reinforcing the need for adequate forecasting and availability of ARVs during a national rollout.^21^

We found that 16% of children who initiated pDTG had one or more ARV switches during follow-up. In most children, these switches involved changes from pDTG to DTG 50 mg, representing the appropriate transition from the lower formulation based on weight. Providers also started a small proportion of children above 20 kg on the 10 mg formulation, which may have been in response to an actual or expected stockout of DTG 50 mg, and changed the child back when there were higher stock levels. Finally, providers may have responded in different ways to weight fluctuations. One might have postponed immediately changing a child’s treatment once they reached a certain weight, while another might have switched a child on and off a drug in response to fluctuating weight.

We found a relatively low pre-pDTG suppression rate, 56.5%, among ART-experienced children who had VL data. The post-pDTG VLS rate increased to 75.1% among ART-experienced children and to 88.9% among newly initiated children. This is consistent with several prior studies in African settings providing evidence of favourable virologic outcomes after initiating DTG-based regimens.^22,23,24^ In our study, older age and having a previously suppressed VL predicted VLS after pDTG-based regimen initiation. On the other hand, we found that almost one in four children who switched to pDTG-based regimen had unsuppressed VL after 5 months on this regimen.

According to the national guidelines, ARV regimens prior to switching included ABC/3TC or AZT/3TC backbones and, after the pDTG national rollout, the guidelines recommended a universal switch of children weighing < 20 kg to ABC/3TC+pDTG regimen. In our study, the majority of children (98.9%) maintained the same pre-switch NRTI backbone after switch, which could contribute to the poor viral suppression rate. The high rates of unsuppressed VL may also reflect a pre-existing resistance to the NRTI backbone that might decrease the effectiveness of DTG.^25^ Given that in our study, 99% of children with unsuppressed VL were ART-experienced children, it is possible that cross-resistance between ABC and other NRTI drugs may partly explain the high proportion of unsuppressed patients after 5 months post switch to a ABC/3TC+pDTG regimen.^26^ Evidence from other studies further emphasise this explanation, indicating that previous exposure to an ART, previous non-suppressed VL, no retention in care, ART duration of more than 24 months and malnutrition contributed to increased rates of virologic failure after a switch to a pDTG-based regimen.^27,28^ Poor adherence is an important contributing factor for poor VLS and ART failure.^29^ A study in Malawi among CLHIV under 20 kg who transitioned to a pDTG-based regimen found that almost half (47.2%) of studied children had poor adherence and it was linked to unsuppressed VL.^30^

We observed that less than half of the children who switched to pDTG had a VL result. Of the 835 children on pDTG for ≥ 5 months, 541 (64.8%) did not have a VL performed, although this figure slightly underestimates VL coverage as we used a shorter duration than the 6-month cut-off per guidelines to increase the number of those contributing to this outcome given the short study period. This low rate is attributable in part to the study’s short follow-up, which was designed with an anticipated pDTG rollout date in January 2022. To collect a VL sample, a child would most likely need to start on pDTG by March 2022 or April 2022. Additionally, newly initiated children were more likely to have fewer months of follow-up, which explains significantly lower VL coverage rates among those just starting treatment (19% compared to 48% for ART-experienced children). However, some of the lower coverage could have been a result of implementation challenges precluding VL monitoring after ART initiation. Insufficient VL testing access has been reported in various HIV-prevalent settings.^31,32^ Although at least yearly VL testing is recommended for HIV care and detection of virological failure,^33^ high costs^34^ and logistical complexities across the various stages of the VL cascade^35^ have been reported in limited-resource settings. Logistical limitations from sample collection, processing and transport, and an extended time for result return can preclude effective VL monitoring.^36^

Therefore, addressing these limitations and improving VL coverage and close VL monitoring is essential to monitor treatment failure. The issue of poor adherence is also worth noting.^29^ Although we did not assess it, we recognise that this might have partly contributed to poor VLS observed in our study.^30^ As such, implementing multidisciplinary strategies and patient-centred approaches to improve adherence are crucial to ensure treatment success. Lastly, addressing issues of the stock management system^37,38^ while improving clinical records documentation^39,40^ are additional strategies to strengthen service provision in the context of a new drug rollout.

There are limitations to this study originating primarily from its use of routine data sources. First, although the study team performed comprehensive data quality assurance activities, we can still not determine if missingness has a service-related justification or was a documentation gap. For instance, one in nine children had no follow-up regimen after switching to or initiating pDTG. Children starting pDTG towards the end of the study period may not have been scheduled to return yet, mainly if they were on a 3-month dispensation schedule and not expected to return until after the study endpoint. They may also have missed their scheduled pick-up visit or become lost to follow-up. Second, the broad study definition of children up to 9 years of age was intended to capture all those with pDTG weight eligibility of < 20 kg, but may have biased the findings towards overestimating eligible children and underestimating transition progress per guidelines. Finally, as noted above, the low VL coverage in our study was exacerbated by the limited VL results available due to the short study follow-up period.

Despite these limitations, our findings present some of the earliest data on real-world national transition to pDTG-based regimens among younger children. This study’s strengths include assessing the uptake of pDTG and comparing virological outcomes pre and post transition among a relatively large sample size of young children while documenting some implementation challenges during a national rollout of pDTG formulation.

## Conclusion

We report on the rollout of the pDTG in a real-world and highly HIV-prevalent setting while comparing post-pDTG virological outcomes of more than a thousand younger children who initiated pDTG. Although most children switched to or initiated pDTG following the national guidelines, programmatic gaps remained. These gaps illuminate several opportunities for further strengthening the rollout of new treatment and formulations and HIV services for children, including more accurate drug forecasting, the need for increasing VL coverage and improved documentation of clinic records.
